# MDM2 inhibitors in myeloid cancers: from basic biology to clinical use in myeloproliferative neoplasms

**DOI:** 10.1038/s41375-026-02975-6

**Published:** 2026-05-05

**Authors:** Haifa K. Al-Ali, Sarah T. Heidel, Francesca Palandri, Florian H. Heidel

**Affiliations:** 1 University Hospital Halle (Saale), Krukenberg Cancer Center Halle, Halle, Germany; 2 Friedrich-Alexander-University Erlangen-Nürnberg, Erlangen, Germany; 3 IRCCS Azienda Ospedaliero-Universitaria di Bologna, Istituto di Ematologia “Seràgnoli”, Bologna, Italy; 4 Hematology, Hemostasis, Oncology and Cell Therapy, Hannover Medical School (MHH), Hannover, Germany

**Keywords:** Myeloproliferative disease, Targeted therapies

## Abstract

Pharmacologic targeting of murine double minute 2 (MDM2) represents one of the most compelling strategies for therapeutic reactivation of wild-type p53 in hematologic malignancies. The MDM2–p53 autoregulatory loop is a central regulator of cellular stress responses, and in myeloid neoplasms—including acute myeloid leukemia (AML) and myeloproliferative neoplasms (MPN)—p53 is frequently retained but functionally suppressed through MDM2 overexpression and oncogenic signaling, notably via JAK–STAT activation. Over the past decade, successive generations of MDM2 inhibitors have translated structural and mechanistic insights into clinical investigation, yielding critical lessons regarding dosing paradigms, hematologic toxicity, biomarker-driven patient selection, and mechanisms of resistance, including TP53-mutant clonal selection. While early phase III trials in AML were negative, recent studies in myelofibrosis demonstrate clinically meaningful spleen, symptom, and molecular responses, supporting disease-modifying potential in TP53–wild-type settings. Adaptive platform designs and rational combinations with JAK inhibitors, BCL-2 antagonists, and interferons have further refined therapeutic strategies. Emerging MDM2 degraders and next-generation agents aim to overcome feedback limitations and improve therapeutic index. This review integrates mechanistic foundations, clinical development, resistance biology, and future directions, highlighting how decades of basic science have reshaped p53 reactivation into a precision therapeutic paradigm in myeloid disease.

## Overview

The pharmacologic targeting of murine double minute 2 (MDM2) has emerged as one of the most conceptually elegant and clinically instructive advances in modern hematologic oncology. Rooted in fundamental discoveries defining the MDM2–TP53 autoregulatory loop as a master regulator of cellular stress responses, this field has progressed from structural and biochemical insight into a clinically actionable strategy for reactivating endogenous tumor suppressor function. In myeloid malignancies—particularly myeloproliferative neoplasms (MPNs) and acute myeloid leukemia (AML)—where TP53 is frequently retained in its wild-type form but functionally restrained, MDM2 inhibition offers a unique opportunity to therapeutically exploit an intact but suppressed p53 pathway. Over the past decade, successive generations of MDM2 inhibitors have translated these principles into the clinic, yielding critical lessons regarding target biology, dosing paradigms, toxicity management, clonal evolution, and biomarker-driven patient selection. At the same time, emerging data have revealed MDM2 as a convergence point for oncogenic signaling, inflammatory cues, and cytokine-driven pathways—most notably JAK–STAT signaling in MPN—thereby positioning MDM2 inhibition not merely as a cytotoxic strategy, but as a potential disease-modifying intervention.

In this review, we integrate mechanistic foundations of the MDM2–p53 axis with the evolution of MDM2 inhibitor development, summarize key clinical experiences across myeloid neoplasms, and discuss resistance mechanisms, safety considerations, and future directions, including rational combination strategies and next-generation approaches such as MDM2 degraders. Together, these insights delineate how decades of basic biology have culminated in a therapeutic paradigm that continues to reshape our understanding of p53 reactivation in myeloid disease.

## Mechanistic foundations: the MDM2-TP53 axis in cellular homeostasis

### Structural and functional architecture of MDM2

MDM2 functions as the principal negative regulator of the tumor suppressor p53 and represents a central signaling hub that integrates structural specificity, post-translational modification, and subcellular trafficking to control cellular fate decisions. The MDM2 oncoprotein is composed of several functionally specialized domains, each contributing to its regulatory versatility. The N-terminal domain harbors the p53-binding interface, which forms a deep hydrophobic cleft accommodating an amphipathic α-helix of p53. High-resolution crystallographic studies demonstrated that three conserved p53 residues—Phe19, Trp23, and Leu26—insert into this pocket, directly masking the p53 transactivation domain and thereby inhibiting transcriptional activation of downstream target genes [1, 2].

**Fig. 1:**
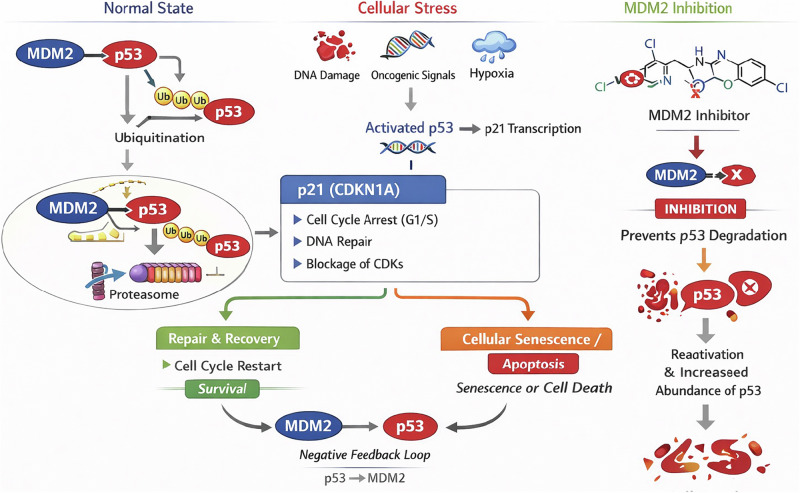
Mechanistic function of the MDM2-p53 axis: regulation under normal conditions, cell stress, and MDM2-inhibitor treatment.

Centrally, MDM2 contains an acidic domain followed by a zinc finger motif that mediates protein–protein interactions beyond p53 binding, particularly with ribosomal proteins such as L5 and L11. These interactions couple MDM2 activity to ribosomal biogenesis and nucleolar stress sensing. Cancer-associated mutations affecting zinc-coordinating cysteine residues (e.g., C305F, C308Y) disrupt ribosomal protein binding, alter MDM2 subcellular localization, and impair efficient p53 degradation, underscoring the importance of this domain in maintaining regulatory fidelity [3]. The C-terminal RING finger domain confers E3 ubiquitin ligase activity, enabling MDM2 to recruit E2 ubiquitin-conjugating enzymes and catalyze mono- and polyubiquitination of lysine residues within the p53 C-terminus, thereby targeting p53 for proteasomal degradation [4]. In addition, MDM2 possesses intrinsic nuclear localization and export signals, allowing it to function as a nuclear–cytoplasmic shuttle that escorts p53 from the nucleus to cytoplasmic proteasomes, adding a spatial layer of control to p53 suppression [5, 6] (Fig. 1).

### The autoregulatory feedback loop

The MDM2–p53 interaction is embedded within one of the most tightly controlled autoregulatory feedback loops in mammalian cells. Under basal conditions, p53 is maintained at low steady-state levels through continuous MDM2-mediated ubiquitination and degradation. Upon cellular stress—such as DNA damage, oncogene activation, hypoxia, or inflammatory signaling—p53 becomes stabilized and transcriptionally active, inducing a broad stress response program. Among its earliest and most robust transcriptional targets is MDM2 itself, establishing a negative feedback loop in which p53 drives expression of its own inhibitor [7, 8]. Newly synthesized MDM2 then attenuates p53 activity through multiple, partially redundant mechanisms: direct binding that occludes the transactivation domain, enforced nuclear export, and ubiquitin-dependent proteasomal degradation (Fig. 1).

This feedback loop ensures rapid termination of p53 signaling once the inciting stress has resolved, thereby preventing inappropriate or prolonged p53 activity that could otherwise trigger excessive cell cycle arrest or apoptosis. Disruption of this equilibrium by post-translational modifications—including phosphorylation, acetylation, or neddylation of either p53 or MDM2—can transiently uncouple the complex, leading to p53 stabilization and selective transcription of downstream effector genes. Among these, CDKN1A (p21) functions as a critical molecular rheostat governing the balance between reversible cell cycle arrest, senescence, and apoptotic commitment (Fig. 1). Sustained p21 induction favors durable growth arrest or senescence, whereas insufficient or transient p21 activation permits engagement of p53-dependent apoptotic programs mediated by BAX, PUMA, and NOXA [9].

### p53-independent functions of MDM2

Beyond its canonical role in p53 regulation, MDM2 exerts multiple p53-independent oncogenic functions that substantially broaden its biological and clinical relevance. MDM2 interacts with key regulators of cell cycle progression and survival, including RB, E2F-1, and FOXO3A, thereby promoting proliferation and apoptosis resistance even in p53-deficient contexts [5, 10]. In addition, MDM2 contributes to tumor angiogenesis through stabilization of VEGF mRNA and functional cooperation with HIF-1α under hypoxic conditions, linking metabolic stress responses to neovascularization.

Emerging evidence further implicates MDM2 in inflammatory and cytokine-driven malignancies. In myeloproliferative neoplasms (MPN), constitutive activation of the JAK–STAT pathway downstream of oncogenic JAK2 signaling has been shown to transcriptionally and post-translationally upregulate MDM2 expression and activity. This reinforces p53 suppression in hematopoietic progenitors and functionally couples inflammatory signaling to impaired tumor suppressor control [11–13]. Thus, MDM2 serves as a convergence point for oncogenic, inflammatory, and stress-response pathways, providing a mechanistic rationale for therapeutic strategies targeting MDM2—either alone or in combination with JAK–STAT inhibition—in MPN and other malignancies characterized by intact but functionally restrained p53.

## Evolution of MDM2 inhibitor development

### First-generation inhibitors: the Nutlin era

The modern era of pharmacological MDM2 inhibition was initiated by the discovery of the nutlin class of small molecules, which provided the first compelling demonstration that a protein–protein interaction as challenging as MDM2–p53 could be therapeutically targeted. Nutlins are cis-imidazoline derivatives rationally designed to occupy the hydrophobic pocket on MDM2 that normally accommodates the p53 transactivation domain, thereby mimicking the key p53 residues Phe19, Trp23, and Leu26 and preventing p53 binding. Early lead compounds such as Nutlin-3a emerged from structure-guided optimization and became widely used chemical probes to validate that selective disruption of the MDM2–p53 interaction results in rapid stabilization of wild-type p53, transcriptional induction of canonical p53 target genes, cell-cycle arrest, and apoptosis in cancer cells [14].

However, first-generation nutlins exhibited pharmacokinetic and metabolic liabilities that precluded direct clinical translation, prompting extensive medicinal chemistry efforts to improve potency, oral bioavailability, and in vivo exposure. These optimization campaigns culminated in the identification of RG7112, a second-generation nutlin derivative and the first MDM2 inhibitor to enter clinical evaluation. RG7112 retained the core imidazoline scaffold but incorporated key structural refinements, including 4,5-dimethyl substitutions that enhanced metabolic stability and binding affinity while preserving the canonical nutlin binding mode within the p53 pocket of MDM2, as confirmed by co-crystal structures [14, 15]. Preclinically, RG7112 demonstrated nanomolar affinity for MDM2, robust activation of p53 signaling, and relevant antitumor activity in xenograft models, particularly in tumors with MDM2 overexpression and intact TP53 [14]. The translation of RG7112 into early-phase clinical trials provided critical proof-of-concept that pharmacological reactivation of p53 is feasible in patients. In relapsed and refractory leukemias, RG7112 induced p53 stabilization, transcriptional upregulation of p53 target genes, and measurable antileukemic activity, including objective responses in a subset of patients with acute myeloid leukemia [16]. At the same time, these studies revealed a narrow therapeutic window. Dose-limiting toxicities were dominated by on-target hematopoietic suppression, most notably thrombocytopenia and neutropenia, reflecting the physiological role of MDM2 in restraining p53 activity during normal hematopoiesis [16]. Gastrointestinal toxicity and significant pill burden further complicated dose escalation. Despite these limitations, the nutlin era was transformative for the field. RG7112 conclusively validated MDM2 as a druggable target in human cancer and established p53 reactivation as a clinically relevant therapeutic strategy. Importantly, the toxicity profile and pharmacological shortcomings of first-generation nutlins provided invaluable mechanistic and translational insights that directly informed the design of next-generation MDM2 inhibitors with improved potency, selectivity, and dosing characteristics, ultimately shaping subsequent waves of MDM2-targeted drug development [17].

### Second-generation advances: idasanutlin

Idasanutlin (RG7388; RO5503781) was developed as a second-generation MDM2 inhibitor with the explicit goal of overcoming the pharmacologic and tolerability limitations observed with first-generation nutlin compounds such as RG7112. While RG7112 established clinical proof-of-concept for p53 reactivation, its requirement for sustained high systemic exposure led to dose-limiting hematologic toxicities that constrained therapeutic flexibility. Idasanutlin emerged from a focused medicinal chemistry effort to improve potency, selectivity, and pharmacokinetic behavior through a strategic scaffold redesign, replacing the cis-imidazoline nutlin core with a structurally distinct pyrrolidine-based chemotype while preserving high-affinity binding to the p53-interaction pocket of MDM2 [18, 19].

This scaffold optimization resulted in a compound with substantially enhanced biochemical potency and cellular activity relative to RG7112, enabling robust stabilization of wildtype p53 and transcriptional induction of canonical p53 target genes at lower concentrations. Importantly, idasanutlin demonstrated improved oral bioavailability and a more favorable pharmacokinetic profile, translating into effective in vivo target engagement at exposures predicted to be better tolerated in the clinical setting [18, 19]. These properties collectively defined idasanutlin as a pharmacologically more tractable MDM2 antagonist, suitable for exploration beyond short-term proof-of-concept dosing.

A key conceptual advance associated with idasanutlin was the recognition that durable antitumor efficacy does not require continuous MDM2 inhibition. Detailed preclinical pharmacokinetic–pharmacodynamic modeling demonstrated temporal disconnect between drug exposure, p53 stabilization, and execution of apoptosis. In vitro pulse-exposure experiments showed that transient idasanutlin treatment was sufficient to irreversibly commit tumor cells to apoptosis, with maximal cell death occurring well after drug washout [19]. Correspondingly, in vivo xenograft studies revealed that intermittent dosing schedules—such as once-weekly or short-course administration—produced tumor growth inhibition comparable to continuous daily dosing despite reduced cumulative drug exposure [19]. These findings had direct translational implications. Intermittent dosing was predicted to preserve antitumor efficacy while mitigating on-target toxicity in normal tissues, particularly the hematopoietic compartment, where physiological MDM2-mediated restraint of p53 is essential for progenitor cell survival. Accordingly, early clinical development of idasanutlin prioritized intermittent schedules within 28-day cycles, representing a departure from the near-continuous exposure paradigm employed for RG7112. This schedule engineering represented a defining feature of second-generation MDM2 inhibitor development, integrating mechanistic insight with pragmatic clinical trial design [16, 19].

Clinically, idasanutlin progressed through multiple phase I studies across solid tumors and hematologic malignancies, including AML, where predictable p53 pathway activation was observed in TP53 wild-type disease. These studies further refined the safety profile of idasanutlin, confirming that hematologic suppression and gastrointestinal effects remained the dominant on-target toxicities, albeit generally manageable with intermittent dosing strategies. Importantly, a pooled concentration–QTc analysis integrating data from three phase I trials demonstrated no exposure-dependent QT prolongation, effectively de-risking a key potential liability for chronic or combination-based administration [20]. The improved therapeutic index of idasanutlin enabled systematic exploration of rational combination strategies, particularly in AML, where MDM2 inhibition was hypothesized to synergize with agents that enhance apoptotic priming or suppress compensatory survival pathways. Although subsequent late-stage clinical outcomes were mixed, idasanutlin nonetheless represents a critical maturation point in the evolution of MDM2 inhibitors. It demonstrated that structure-guided medicinal chemistry, coupled with mechanism-informed dosing strategies, could substantially improve the clinical tractability of p53 reactivation as a therapeutic approach. In doing so, idasanutlin established key principles that continue to inform the development of newer MDM2 antagonists and p53-pathway–targeted combinations in leukemia and beyond [16, 18, 19].

### Third-generation potency: siremadlin and navtemadlin

The current generation of MDM2 inhibitors represents the culmination of long-term investigation of structural biology, medicinal chemistry, and clinical experience. Siremadlin (HDM201, NVP-HDM201) exhibits picomolar affinity for MDM2 with greater than 10,000-fold selectivity over MDM4. This selectivity profile has enabled more targeted therapeutic approaches with reduced off-target effects [21].

Clinical development of siremadlin has focused primarily on solid tumors, with phase II studies in various malignancies including liposarcoma and myelofibrosis (MF). Notably, siremadlin development in AML was discontinued, likely due to challenges with hematologic toxicity. However, recent studies have shown promising activity in chronic lymphocytic leukemia with TP53 wild-type status.

Navtemadlin (KRT-232, APG-115) represents another best-in-class MDM2 inhibitor with distinct pharmacological properties. This compound has demonstrated remarkable clinical activity in MF leading to its evaluation in the phase III BOREAS trial [22]. The success of navtemadlin in MF represents a watershed moment for the field, proving that MDM2 inhibition can achieve clinically meaningful outcomes in hematologic malignancies.

## Pioneering MDM2 research in myeloid neoplasms

### Foundational discoveries in MPN biology

Ronald Hoffman’s work has been instrumental in defining dysregulated p53 signaling as a central and therapeutically exploitable vulnerability in myeloproliferative neoplasms (MPNs). Through integrated mechanistic and translational studies, a framework was established in which disease-initiating hematopoietic stem and progenitor cells—rather than mature effector populations—were identified as critical therapeutic targets. MDM2 overexpression was identified in CD34⁺ cells from patients with MPNs, with the highest levels observed in primary MF compared with polycythemia vera (PV) and normal controls. Notably, this occurred in the context of largely intact TP53, indicating functional suppression of p53 rather than genetic inactivation. These findings positioned MDM2 as both a biomarker of disease progression and a rational therapeutic target in chronic-phase MPNs. Pharmacologic antagonism of MDM2 was shown to selectively restore p53 activity in malignant progenitors [23]. Nutlin-class compounds preferentially suppressed erythroid and myeloid colony formation derived from PV CD34⁺ cells while largely sparing normal hematopoiesis. Genotyping of individual colonies demonstrated selective depletion of JAK2V617F-positive progenitors, particularly heterozygous clones, consistent with an increased dependency of mutant cells on MDM2-mediated p53 repression. These effects were associated with p53 stabilization and induction of downstream cell-cycle and apoptotic regulators, including p21, PUMA, and BAX. A mechanistic rationale for combination therapy emerged from studies combining MDM2 antagonists with pegylated interferon-α2a [23]. Complementary activation of the p53 pathway was observed, with interferon enhancing p53 transcription via STAT1 and p38 MAPK signaling and MDM2 inhibition preventing p53 degradation. At subtherapeutic doses, this combination synergistically enhanced apoptosis in MPN CD34⁺ cells and reduced JAK2V617F-positive colony formation, supporting a clonal depletion strategy rather than cytoreduction alone. These preclinical findings were translated into clinical investigation with oral MDM2 inhibitors [24]. In an investigator-initiated phase 1 study of idasanutlin in patients with high-risk, treatment-refractory PV and essential thrombocythemia, on-target p53 activation, hematologic and symptomatic responses, and reductions in JAK2V617F allele burden were observed, with enhanced activity following the addition of low-dose interferon-α2a. A biomarker-driven translational strategy was central to this work. Pharmacodynamic readouts, including p53 target gene induction, serial assessment of JAK2V617F allele burden, and bone marrow histopathology, enabled evaluation of biological response beyond peripheral blood count normalization. In parallel, patient-derived xenograft models were developed to interrogate therapeutic effects on hierarchically organized MPN stem cell populations, providing critical preclinical platforms for identifying the cellular compartments required for durable disease modification.

Collectively, these studies established functional p53 suppression as a defining feature of chronic-phase MPNs and provided a translational blueprint for stem cell–directed therapeutic strategies aimed at achieving sustained clonal and histologic responses.

The combination studies pioneered by Hoffman and colleagues established a critical preclinical foundation for rational disease-modifying strategies in MPNs. Using primary CD34⁺ cells from patients with PV and primary MF, they demonstrated that the orally bioavailable MDM2 antagonist RG7112, particularly when combined with pegylated interferon-α2a, exerted potent and selective effects on malignant hematopoietic progenitor and stem cell compartments [25]. At sub-optimal concentrations that spared normal hematopoiesis, this combination significantly reduced CFU-GM and BFU-E colony formation and preferentially depleted JAK2V617F-positive progenitors, with a pronounced reduction in heterozygous mutant colonies. Mechanistically, these effects were linked to disruption of the p53–MDM2 interaction, resulting in restoration of p53 signaling and induction of apoptosis selectively in MPN CD34⁺ cells, as evidenced by increased Annexin V positivity, caspase-3 activation, and upregulation of canonical p53 target genes including p21, PUMA, and Bax. Importantly, functional stem cell assays revealed that short-term ex vivo exposure to RG7112 and pegylated interferon-α2a markedly impaired the ability of MPN CD34⁺ cells to engraft and repopulate immunodeficient NSG mice, accompanied by a substantial reduction in JAK2V617F allele burden in vivo. These data provided direct evidence that combined p53 pathway reactivation and interferon signaling can target disease-initiating cells in MPNs, thereby offering a strong biological rationale for low-dose combination approaches aimed at clonal depletion rather than mere cytoreduction—an insight that has subsequently informed the translation of MDM2 inhibitor–based combinations into early-phase clinical investigation.

### Contemporary clinical developments

Third-generation MDM2 targeting in MPNs is increasingly moving from proof-of-concept inhibitors toward more potent, schedule-flexible agents designed to deepen spleen and symptom responses and potentially modify disease biology. Siremadlin (HDM201) is being explored in ruxolitinib-based combination strategies within adaptive platform frameworks (e.g., ADORE), where early signals suggest that pairing p53 reactivation with JAK inhibition can enhance clinical activity in MF. In parallel, navtemadlin (KRT-232) has advanced into late-stage MF development, including monotherapy registrational efforts in JAK inhibitor-relapsed/refractory disease (BOREAS) and the global phase 3 POIESIS trial, which evaluates navtemadlin as an add-on to ruxolitinib specifically in JAK inhibitor–naïve patients with a suboptimal ruxolitinib response—an architecture aligned with real-world clinical decision making. Looking ahead, next-generation MDM2 inhibitors and emerging MDM2-directed proteolysis-targeting chimeras (PROTACs) aim to improve the therapeutic index by moving from reversible blockade to targeted MDM2 elimination, potentially delivering more durable p53 pathway reactivation and offering new combination options as these modalities enter early clinical translation.

### ADORE: platform innovation in clinical trial design to siremadlin combination success

The ADORE (Assessment of Dual Combinations in Ruxolitinib Non-optimal Responders with Myelofibrosis) study (NCT04097821) represents an important evolution in clinical trial design for rare hematologic cancers. By implementing a phase 1b/2 open, adaptive platform architecture, ADORE addressed the central challenge of testing multiple investigational therapies in a population of patients with myelofibrosis (MF) who exhibit suboptimal responses to ruxolitinib (Table 1). Instead of evaluating candidate drugs sequentially in separate, traditional early-phase trials—each requiring its own enrollment, infrastructure, and control arm—ADORE embedded ruxolitinib as the therapeutic backbone and permitted simultaneous, harmonized assessment of five mechanistically distinct agents: siremadlin, rineterkib, sabatolimab, crizanlizumab, and NIS793 [26]. This integrated design increased the efficiency and scientific yield of clinical development, enabling rapid prioritization of promising combinations while minimizing the burden on a limited patient pool.

**Table 1:** Clinically developed MDM2-targeting compounds in myeloid cancers.

Compound	Type	Trial (ID & Acronym)	Phase	Clinical Outcome / Endpoints (Brief)	Citation
Siremadlin (HDM201)	Small Molecule Inhibitor	**NCT04097821** *(ADORE)*	Phase 1b/2	**Efficacy signals observed**. Open-label platform study in R/R Myelofibrosis. Ruxolitinib + Siremadlin (30 mg) arm showed the most robust Spleen Volume Reduction (SVR) among 5 combo arms. SVR ≥ 35% observed in 60% of evaluable patients at Wk 24.	[26]
Navtemadlin (KRT-232)	Small Molecule Inhibitor	**NCT06479135** *(POIESIS)*	Phase 3	**Ongoing/Active**. Evaluating Navtemadlin as “add-on” to Ruxolitinib in JAKi-naïve MF patients with **suboptimal response** (SVR < 35% & TSS < 50%) after run-in. Primary endpoints: SVR35 and TSS50 at Wk 24 vs placebo add-on.	[38]
Idasanutlin (RG7388)	Small Molecule Inhibitor	**NCT02545283** *(MIRROS)*	Phase 3	**Primary endpoint (OS) not met**. Trial in R/R AML (Idasanutlin + Cytarabine) terminated for futility. No significant improvement in Overall Survival compared to placebo arm; increased GI toxicity observed.	[55, 56]
Navtemadlin (KRT-232)	Small Molecule Inhibitor	**NCT03930732** *(BOREAS)*	Phase 3	**Primary endpoint (SVR35) not met**. In R/R Myelofibrosis (post-JAKi), SVR35 was 15% (Navtemadlin) vs 5% (BAT), which did not reach statistical significance (p = 0.08). TSS50 improved (24% vs 12%, p = 0.05).	[27]
Siremadlin (HDM201)	Small Molecule Inhibitor	**NCT02143635** *(Study X2101)*	Phase 1b	**Safety/RP2D established**. First-in-human study in AML/MDS. Monotherapy ORR ~ 20% in R/R AML. Identified intermittent dosing (Day 1, 8 q4w) to manage bone marrow suppression.	[21]
Milademetan (DS-3032b)	Small Molecule Inhibitor	**NCT03634228** *(N/A)*	Phase 1b	**Safety profile defined**. Combo with LDAC ± Venetoclax in R/R AML showed modest efficacy (ORR 13%). Dose-limiting GI toxicity and limited durability in heavily pre-treated patients.	[34]
Alrizomadlin (APG-115)	Small Molecule Inhibitor	**NCT03611868** *(N/A)*	Phase 2	**Efficacy signals observed**. Evaluated with Azacitidine in R/R AML/MDS. Preliminary data showed p53 activation and synergism in HMA-refractory patients.	[57]
Goputamig (AMG 232)	Small Molecule Inhibitor	**NCT02016729** *(N/A)*	Phase 1b	**Safety/Efficacy signals established**. Monotherapy in *TP53*-WT AML showed 31% response rate (4/13 patients). On-target biomarker induction (MIC-1) confirmed.	[58]
KT-253	Heterobifunctional Degrader (PROTAC)	**NCT05775406** *(N/A)*	Phase 1	**Pharmacodynamic proof-of-concept**. First-in-class degrader. Achieved rapid p53 upregulation (GDF-15) without the neutropenia/thrombocytopenia typical of inhibitors in early cohorts.	[54, 59, 60]

Among the 44 patients enrolled in part 1, the largest cohort received the combination of ruxolitinib and the MDM2 inhibitor siremadlin (*n* = 23). These patients had a median age of 68 years and predominantly primary MF (65.2%), with substantial prior exposure to ruxolitinib (median 67.9 weeks; 165.1 weeks in the 30 mg siremadlin subgroup). Molecular profiling at baseline revealed JAK2V617F mutations in 14 patients, CALR mutations in 5, and high-risk mutations in ASXL1, SRSF2, EZH2, IDH1/2, or U2AF1 in 10 patients, reflecting the mutational heterogeneity characteristic of advanced MF. Siremadlin, a potent MDM2 inhibitor, is designed to reactivate p53-mediated apoptosis and thereby promote clonal regression. Preclinical data have suggested that activating the p53 pathway in combination with JAK inhibition may shift the disease biology toward deeper and potentially disease-modifying responses, providing the mechanistic rationale for this combination.

The safety profile observed with ruxolitinib plus siremadlin was consistent with the expected effects of MDM2 inhibition and the underlying hematopoietic fragility of the MF population. Hematologic toxicities were the most frequent adverse events, including grade ≥3 anemia (60.9%), thrombocytopenia (47.8%), and neutropenia (47.8%). Gastrointestinal adverse events such as nausea and diarrhea were common but typically low grade. Notably, gastrointestinal toxicity appeared less pronounced than that reported with other MDM2 inhibitors, although direct comparisons require dedicated evaluation. Dose-limiting toxicities occurred primarily in the 40 mg cohort, allowing clear establishment of 30 mg once daily on days 1–5 of a 28-day cycle as the recommended phase 2 dose. Despite the expected cytopenias, the overall safety of the combination was manageable, and no correlation was observed between increased siremadlin exposure and the frequency or severity of adverse events.

In terms of clinical activity, the ruxolitinib–siremadlin combination emerged as the most active regimen evaluated within the platform. At week 24, 30.4% of patients achieved a spleen volume reduction of at least 35% (SVR35), with a notable 60% response rate in the 30 mg siremadlin cohort—the highest proportion among all arms in ADORE. Median spleen volume reductions reached −22.8% overall and −38.7% in the 30 mg cohort, representing meaningful additional benefit in a population already receiving and inadequately responding to ruxolitinib. Symptom improvements paralleled these anatomical responses: 21.7% of patients experienced ≥50% reduction in total symptom score (TSS50) at week 24, including individuals across all dose cohorts. These symptomatic and volumetric responses are particularly compelling given the prolonged ruxolitinib exposure and generally modest symptom burden at baseline.

Translational endpoints further supported the biological activity of siremadlin. Reductions in JAK2V617F allele burden were observed, with a median decrease of −12.6% at week 24 and deeper declines in some patients, including those with concomitant spleen responses. Several CALR-mutant patients similarly demonstrated reductions in variant allele frequency. These findings align with the known but typically slow and modest allele burden reductions associated with long-term ruxolitinib monotherapy and suggest that MDM2 inhibition may lead on an increment of molecular responses. Pharmacodynamic confirmation of p53 pathway engagement was demonstrated across all siremadlin dose levels by increased circulating GDF-15 concentrations, with the strongest induction observed in the 30 mg cohort.

An important exploratory observation was the emergence of low-variant allele frequency TP53 mutations (2–8%) in six patients receiving siremadlin. These mutations, located in the DNA-binding domain and consistent with loss-of-function changes, mirror findings from other studies of MDM2 inhibitors, such as idasanutlin in PV. Although the clinical significance of these mutations remains uncertain due to their low abundance and the absence of detailed single-cell analyses, they underscore the need for careful future assessment of clonal dynamics, particularly given the known prognostic implications of TP53 alterations in myeloid neoplasia. Additional mutations were detected during therapy in other genes, but their relevance could not be fully established.

Although early termination of study enrollment limited the full realization of the platform design—particularly the transition to parts 2 and 3—the available data clearly illustrate the feasibility and scientific value of the ADORE approach. Within the context of this adaptive platform, the combination of ruxolitinib and siremadlin, particularly at the 30 mg dose level, demonstrated the most consistent and compelling evidence of clinical benefit, supported by molecular and pharmacodynamic activity and a manageable toxicity profile.

Taken together, the ADORE results position siremadlin as a particularly promising partner for ruxolitinib in MF. The combination not only improved spleen size and symptoms in patients with established suboptimal responses to JAK inhibition but also provided initial signals of disease modification through reductions in driver mutation allele burdens and targeted activation of p53 signaling. Despite the study’s early termination, the unexplored potential of siremadlin in MPNs remains considerable. Future investigations could examine its role in earlier disease stages, in combination or sequence with other emerging disease-modifying therapies, and with deeper genomic characterization to clarify its influence on clonal evolution. As such, siremadlin represents an important candidate for next-generation therapeutic strategies in MF and the broader spectrum of MPNs, warranting continued and rigorous clinical exploration.

### BOREAS: phase III validation of MDM2 inhibition

The BOREAS trial (NCT03662126) represents the definitive evaluation of MDM2 inhibition in MF, comparing navtemadlin monotherapy to best available therapy (BAT) in patients with relapsed/refractory disease following JAK inhibitor treatment [22]. This randomized, multicenter, global phase III study enrolled 183 patients with TP53 wild-type MF who were refractory or had relapsed on prior JAK inhibitor therapy [27] (Table 1). Patients were randomly assigned 2:1 to receive navtemadlin 240 mg for 7 days in a 28-day cycle (with 21 days drug holiday) or BAT including monotherapy or combinations: hydroxyurea, chemotherapy, IMiDs, and supportive care; JAK inhibitors were excluded. Anti-diarrhea prophylaxis was administered for the first two cycles and anti-nausea prophylaxis was given with every cycle for the seven days of navtemadlin treatment.The primary endpoint was SVR35 at week 24, with secondary endpoints including TSS50, overall survival, and progression-free survival.. Navtemadlin was safe and well-tolerated. Most common hematologic grade 3/4 treatment-emergent adverse events were thrombocytopenia (37% vs 21%), anemia (29% vs 25%), and neutropenia (24% vs 12%) with navtemadlin vs BAT, respectively. Gastrointestinal grade 3/4 adverse events were diarrhea (5% vs 2%), nausea (3% vs 0%), and vomiting (2% vs 0%) with navtemadlin vs BAT, respectively.

In this study, navtemadlin demonstrated clinically relevant efficacy in patients, refractory or relapsed on prior JAK inhibitor therapy. The rate of SVR35 and TSS50 at week 24 was three-fold [15% (18/123) vs 5% (3/60)] and two-fold [24% (30/123) vs 12% (7/60)] higher with navtemadlin vs BAT.

Notably, navtemadlin has the potential to improve biomarkers of disease burden in pts who were refractory or had relapsed on prior JAK inhibitor therapy. This is suggestive of anti-clonal activity and disease modification. In the BOREAS study, the on-target biological activity of navtemadlin was reflected by substantial decreases in peripheral blood CD34+ cell counts, MF driver variant allele frequency (VAF) ≥ 50%, and selected serum inflammatory cytokine levels such as TNF-alpha, IL-6, and CRP. Furthermore, these changes in disease biomarkers with navtemadlin treatment were significantly correlated with the magnitude of SVR; demonstrating an effect between navtemadlin-induced disease modification and response Finally, bone marrow fibrosis grade was reported to improve by one or more in 47% (31/66) of navtemadlin treated pattients who had paired bone marrow biopsies at baseline and Week 24 [28]. Similar to what has been reported in the phase II trial with navtemadlin monotherapy in patients, refractory or relapsed on prior JAK inhibitor therapy, the translational findings of the phase III BOREAS study indicate that altering the underlying biological mechanisms driving MF through a therapeutic intervention could translate into a demonstrable clinical benefit [29].

Taken together, the biomarker analyses revealed encouraging signals suggesting that navtemadlin may target disease-driving cells and alter disease progression. While detailed efficacy results await final publication, the presented data at the 66th ASH Annual Meeting support the disease-modifying potential of MDM2 inhibition in MF.

### Mechanistic insights from recent clinical experience

#### Resistance mechanisms and clonal evolution

Recent clinical experience has provided important insights into resistance mechanisms for MDM2 inhibitors. A concerning observation is the emergence of TP53-altered clones following MDM2 inhibitor treatment. In a case report, a patient with MDM2-amplified adenoid cystic carcinoma developed persistent pancytopenia associated with 20 distinct pathogenic TP53 mutations in peripheral blood and bone marrow following MDM2 inhibitor treatment [30]. This phenomenon, termed MDM2 inhibitor-associated clonal hematopoiesis, represents a previously unrecognized toxicity that may confer risk for subsequent myeloid malignancy development. Plasma TP53 mutations were detected in multiple patients, with the number of mutations correlating strongly with duration of treatment [30]. These findings underscore the need for careful monitoring of patients receiving MDM2 inhibitors and may influence future clinical trial designs.

Laboratory studies have confirmed that MDM2 inhibition can accelerate the selection of TP53-mutant clones in vitro. Long-term culture of AML cells with increasing concentrations of idasanutlin resulted in the emergence of resistant clones harboring TP53 mutations. Single-cell analysis of MPN patient samples has further refined this evolutionary model, revealing that MDM2 inhibition does not merely induce mutagenesis but exerts profound selective pressure on pre-existing, rare TP53-mutated subclones [31]. In these competitive assays, TP53-mutant cells demonstrated a distinct fitness advantage over wild-type counterparts, rapidly dominating the clonal architecture under therapeutic stress. This “clonal sweeping” effect suggests that the duration of MDM2 inhibitor exposure is a critical variable, as prolonged suppression of the TP53-wild-type major clone creates a permissive niche for the expansion of resistant minor subclones [31, 32]. Notably, extended follow-up consistent across both siremadlin and navtemadlin trials indicates that this expansion of minor TP53-mutated subclones is generally reversible upon treatment discontinuation and has not been definitively linked to an increased incidence of overt secondary leukemia development to date [33–35].

Beyond genetic evolution, adaptive resistance mechanisms independent of TP53 status have also been identified. Upregulation of anti-apoptotic BCL-2 family members, particularly MCL-1, serves as a compensatory survival pathway in response to p53 activation [36]. Mechanistically, MDM2 inhibition can trigger a feedback loop involving the stabilization of MCL-1, thereby raising the apoptotic threshold and limiting the cytotoxic efficacy of monotherapy [37]. Furthermore, recent data describe an inflammatory resistance axis driven by a CEBPB/IL-1β/TNF-α feedback loop in monocytic AML subtypes, which facilitates leukemia cell survival despite robust p53 activation [32]. These mechanistic insights have important implications for understanding the long-term safety profile of MDM2 inhibitors and provide a strong rationale for combination strategies—such as concurrent BCL-2 or MCL-1 inhibition—to mitigate both genetic and non-genetic resistance development.

#### Pharmacodynamic biomarkers and patient selection

The clinical development of MDM2 inhibitors has been tightly coupled to the identification and validation of robust pharmacodynamic biomarkers that reliably reflect on-target engagement of the p53 pathway and enable rational patient selection. Among these, circulating growth differentiation factor 15 (GDF-15), also referred to as macrophage inhibitory cytokine-1 (MIC-1), has emerged as a particularly informative and practical biomarker. GDF-15 is a direct transcriptional target of p53, and its rapid, dose-dependent induction following MDM2 inhibition provides compelling evidence of functional p53 reactivation in vivo. In early clinical studies of the first-generation MDM2 antagonist RG7112, increases in serum GDF-15 were consistently observed and correlated with systemic drug exposure, confirming target engagement across hematologic malignancies [16]. Similar pharmacodynamic signatures have since been reproduced with next-generation MDM2 inhibitors, including idasanutlin and siremadlin, establishing GDF-15 induction as a class-defining biomarker for MDM2 antagonism [24]. Beyond soluble biomarkers, modulation of intracellular p53 downstream effectors provides additional mechanistic resolution. Transcriptional upregulation of canonical p53 target genes involved in cell-cycle arrest (e.g., CDKN1A/p21), apoptosis (e.g., BBC3/PUMA, BAX), and stress responses has been documented in leukemic blasts and CD34⁺ progenitor populations following MDM2 inhibitor exposure. In the RG7112 phase I study, induction of p53 target gene expression and accumulation of p53 protein were restricted to TP53 wild-type cells, underscoring the biological specificity of this therapeutic approach [16]. Complementary analyses demonstrated early increases in apoptotic markers, including annexin V positivity and cleavage of pro-apoptotic proteins, supporting their potential role as early indicators of therapeutic response prior to overt clinical benefit. Patient selection strategies for MDM2 inhibitor trials have therefore evolved toward a biomarker-driven framework centered on preservation of functional p53 signaling. Across tumor types, TP53 wild-type status has emerged as a foundational inclusion criterion, reflecting the mechanistic requirement for intact p53 transcriptional activity. Importantly, integrated genomic analyses have further refined this paradigm by demonstrating a reciprocal relationship between TP53 mutations and MDM2 copy number alterations. In AML and MPNs, TP53 mutations occur at significantly lower frequency in tumors harboring MDM2 overexpression or amplification, suggesting alternative, mutually exclusive mechanisms of p53 pathway suppression [16]. This mutual exclusivity has directly informed eligibility criteria in subsequent clinical trials, enriching study populations for patients most likely to derive benefit from MDM2 inhibition. The relevance of these selection principles has been reinforced in chronic myeloid neoplasms. In patients with PV and essential thrombocythemia treated with idasanutlin, pharmacodynamic activation of the p53 pathway—evidenced by GDF-15 induction and depletion of malignant CD34⁺ stem and progenitor cells—was observed predominantly in TP53 wild-type disease, and clinical responses were accompanied by molecular remissions and reductions in driver mutation allele burden [24]. Likewise, decreases in peripheral blood CD34+ cell counts, MF driver VAF, and inflammatory cytokine levels were translated into a demonstrable clinical benefit in patients treated with navtemalin [22, 27]. These findings support the concept that pharmacodynamic biomarkers not only confirm target engagement but may also serve as surrogates for disease-modifying activity. More recent combination studies have extended these insights into advanced MF. In the ADORE platform trial, treatment with siremadlin plus ruxolitinib resulted in consistent elevations of circulating GDF-15, confirming sustained p53 pathway activation in the combination setting, alongside reductions in spleen volume and JAK2V617F allele burden in responding patients [26].

These observations have directly shaped the design of the ongoing phase 3 POIESIS trial (NCT06479135), which prospectively restricts enrollment to TP53 wild-type patients and integrates biomarker-driven endpoints to isolate the clinical contribution of add-on navtemadlin [38]. Collectively, these pharmacodynamic and genomic insights illustrate how biomarker-guided development has transformed MDM2 inhibition from a broadly applied cytotoxic strategy into a precision medicine approach. The development of robust pharmacodynamic biomarkers has been crucial for optimizing MDM2 inhibitor therapy. The demonstration of on-target p53 pathway activation through increased GDF-15 levels provides a practical biomarker for confirming drug activity. Additionally, changes in pro-apoptotic proteins and cell cycle markers can serve as indicators of therapeutic response.

Patient selection strategies have evolved to focus on TP53 wild-type tumors with evidence of MDM2 overexpression or amplification. The mutual exclusivity analysis demonstrating that TP53 mutations decrease with increasing MDM2 copy number has informed enrollment criteria for current clinical trials. These biomarker-driven approaches represent a crucial advance toward precision medicine applications of MDM2 inhibitors.

### Safety considerations and toxicity management

#### Hematologic toxicity profiles

Hematologic toxicity represents the principal dose-limiting adverse effect of MDM2 inhibitors and has been consistently observed across all clinical programs targeting p53 reactivation. This class effect reflects the essential physiological role of MDM2 in restraining p53 activity within normal hematopoietic stem and progenitor cells, where unchecked p53 activation leads to cell-cycle arrest and apoptosis. Across early- and late-phase clinical trials, thrombocytopenia, neutropenia, and anemia have emerged as the most frequent grade 3–4 toxicities, often necessitating dose interruptions, reductions, or discontinuation of therapy [16]. Among these, thrombocytopenia has been the dominant and earliest dose-limiting toxicity, typically manifesting within the first one to two treatment cycles and correlating closely with cumulative drug exposure.

Comparative analyses of multiple MDM2 inhibitor trials, encompassing diverse chemical scaffolds and tumor indications, have demonstrated remarkably consistent patterns of myelosuppression, indicating that hematologic toxicity is largely mechanism-driven rather than compound-specific [19]. Importantly, these observations have directly shaped modern dosing paradigms. Preclinical and clinical pharmacokinetic–pharmacodynamic modeling revealed that transient p53 activation is sufficient to induce apoptosis in malignant cells, whereas normal hematopoietic cells demonstrate more rapid recovery following drug withdrawal. This differential recovery kinetics provided the rationale for intermittent dosing strategies incorporating treatment holidays to permit marrow recovery while preserving antitumor efficacy [19].

Clinically, intermittent schedules—such as weekly, biweekly, or short-course dosing within 28-day cycles—have substantially improved tolerability without compromising pharmacodynamic target engagement. These advances have enabled continued development of next-generation MDM2 inhibitors and facilitated combination strategies, particularly in hematologic malignancies, where careful balancing of efficacy and marrow toxicity remains paramount [20].

#### Gastrointestinal and other non-hematologic toxicities

Beyond hematologic effects, MDM2 inhibitors consistently induce gastrointestinal (GI) toxicities, most commonly nausea, vomiting, diarrhea, and decreased appetite, which represent the dominant non-hematologic adverse events reported across early- and late-phase clinical trials. These toxicities are generally dose- and schedule-dependent and are widely regarded as on-target effects of p53 reactivation in rapidly proliferating gastrointestinal epithelial cells, where p53 plays a central role in regulating cell-cycle arrest and apoptosis [16, 39, 40]. In early nutlin-based compounds, such as RG7112, GI toxicity often limited dose escalation and continuous dosing; however, second- and third-generation inhibitors have benefited from improved pharmacokinetics and intermittent dosing schedules that substantially mitigate severity [19, 20].

Prophylactic antiemetic regimens, antidiarrheal agents, and short-course dosing have become standard supportive strategies and have enabled sustained drug exposure in later-stage trials. Additional non-hematologic toxicities include fatigue, asthenia, anorexia, and occasional dermatologic manifestations, such as rash or pruritus, which are typically low-grade and reversible with treatment interruption or dose modification [16, 41, 42]. Importantly, comprehensive cardiac safety evaluations have largely excluded clinically meaningful QT prolongation for newer MDM2 inhibitors, alleviating an early concern for chronic administration [20]. Overall, while non-hematologic toxicities remain a defining class effect, ongoing optimization of compound selectivity, dosing schedules, and supportive care has rendered the safety profile increasingly manageable, supporting continued clinical development of MDM2-targeted therapies.

### Future directions and combination strategies

#### Rational combination approaches

The future of MDM2 inhibitor development lies in rational combination strategies that can enhance efficacy while managing toxicity. The success of the ruxolitinib plus siremadlin combination in ADORE provides a template for developing other synergistic combinations. Potential combination partners include JAK inhibitors, BCL-2 inhibitors, CDK4/6 inhibitors, and immune checkpoint inhibitors. The biological rationale for combining MDM2 inhibitors with other targeted agents is strong. MDM2 inhibition activates p53-dependent apoptotic pathways, which can be enhanced by BCL-2 inhibition. Similarly, the cell cycle effects of MDM2 inhibition may synergize with CDK4/6 inhibitors. These combinations require careful dose optimization to balance efficacy and toxicity.

#### JAK inhibitor combinations

In nonclinical studies, Clevenger et al. showed that navtemadlin and ruxolitinib synergistically enhanced apoptosis in MF patient-derived CD34+ progenitor cells by inhibition of p21-mediated cell-cycle arrest [43]. Consistent with the nonclinical results, clinical data of the phase 1b/2 KRT-232-109 trial of navtemadlin add-on to ruxolitinib in TP53WT patients with MF who had a suboptimal response to ruxolitinib evidenced the robust efficacy and synergy of navtemadlin add-on to ruxolitinib with 32% (6/19) of patients achieving SVR35 (primary endpoint) and TSS50 (key secondary endpoint) at week 24. Notably, patients had received ruxolitinib for a long period of time prior to study entry, with most on doses ≥15 mg twice daily, and yet they were still unable to achieve an optimal response. Ruxolitinib dose increases were not permitted, confirming that the observed clinical responses were attributable to navtemadlin [44]. The results of this phase 1b/2 study supported the design of the phase III POIESIS trial.

The global registrational **POIESIS study (NCT06479135)** employs a rigorously structured and clinically pragmatic trial design to evaluate navtemadlin as add-on therapy to ruxolitinib in JAK inhibitor–naïve patients with MF who fail to achieve an optimal response to frontline JAK inhibition. Central to the POIESIS architecture is a prolonged ruxolitinib monotherapy run-in phase that mirrors real-world treatment practice, in which ruxolitinib is initiated, titrated, and stabilized before consideration of additional therapy [45]. Approximately 600 patients receive ruxolitinib alone for 18–24 weeks, allowing for identification of distinct clinical response phenotypes under standardized exposure. Patients achieving optimal spleen or symptom responses, as well as those with primary ruxolitinib-refractory or progressive disease, are discontinued, thereby enriching the randomized population for individuals with a clearly defined suboptimal response despite stable ruxolitinib dosing. This approach minimizes heterogeneity and isolates a population with ongoing unmet clinical need. Eligibility for randomization additionally requires confirmed TP53 wild-type status, ensuring preservation of navtemadlin’s mechanistic dependence on intact p53 signaling. By randomizing only suboptimal responders to navtemadlin or placebo on a background of continued ruxolitinib, POIESIS is uniquely positioned to isolate the incremental clinical contribution of MDM2 inhibition, while maintaining the integrity of established endpoints for spleen volume reduction, symptom improvement, and long-term outcomes, including progression-free and overall survival.

#### Immunotherapy combinations

The combination of MDM2 inhibitors with immunotherapeutic agents has emerged as a potential therapeutic strategy, supported by preclinical evidence demonstrating that MDM2 inhibition enhances antitumor immunity through multiple mechanisms including upregulation of IL-15 and MHC class II production and improved T cell infiltration in tumor models [46]. The most extensively studied combinations involve immune checkpoint inhibitors, particularly PD-1/PD-L1 blockade. Clinical evaluation of the MDM2/p53 antagonist alrizomadlin (APG-115) in combination with pembrolizumab has shown preliminary activity in patients with advanced, immunotherapy-refractory solid tumors, including melanoma, NSCLC, and urothelial cancer, with objective responses observed across multiple cohorts [47]. Brigimadlin (BI-907828), another potent MDM2-p53 antagonist, has demonstrated antitumor activity in patients with advanced MDM2-amplified biliary tract cancer, including partial responses in a series of treated patients, supporting ongoing investigation of MDM2 inhibition with or without checkpoint blockade [40]. An ongoing phase I/II study is evaluating milademetan in combination with atezolizumab to better define tolerability and clinical activity in solid tumors (trial design reported; clinical data pending publication).

The combination of MDM2 inhibitors with interferons has shown particular promise in MPNs, where RG7112 combined with pegylated interferon-α2a preferentially targeted JAK2V617F-positive hematopoietic progenitor cells and reduced mutant allele burden in preclinical xenograft models. MDM2 inhibitors have also been explored with CAR-T cell therapy based on their ability to modulate the immunogenicity of cancer cells and influence NKG2D ligand expression, potentially enhancing CAR-T efficacy, although clinical evidence remains to be published [48]. The combination with oncolytic adenoviruses demonstrated synergistic cytotoxicity in mesothelioma models, with MDM2 inhibitors facilitating viral progeny production and amplifying p53-mediated apoptotic pathways [49, 50]. A vaccine-based approach combining an MDM2-derived peptide with Nutlin-3 showed that peptide-specific CD4 + T cells can kill tumor cells via granzyme B, with MDM2 inhibition augmenting responses via CIITA upregulation [48]. Dual MDM2/MDMX inhibitors such as ALRN-6924 have demonstrated immunomodulatory properties and increased T cell infiltration when paired with checkpoint blockade in preclinical and early clinical studies [51].

Notably, MDM2 amplification has been identified as a mechanism associated with hyperprogression during checkpoint inhibitor therapy in some tumor types, suggesting that combining MDM2 inhibition with immunotherapy might mitigate accelerated tumor growth in MDM2-amplified settings. Clinical development has been guided by predictive biomarkers, with wild-type TP53 being essential for activity and high-level MDM2 amplification correlating with enhanced responses in MDM2-targeted therapy trials [52]. Despite encouraging preliminary data, challenges remain including on-target hematologic toxicities requiring intermittent dosing and the discontinuation of some investigational agents despite promising phase I activity. Current active trials continue to refine optimal combinations, dosing strategies, and patient selection criteria for MDM2 inhibitor-based immunotherapy regimens across multiple tumor types.

#### Next-generation MDM2 inhibitors and PROTACs

A major limitation of “first-wave” MDM2–p53 antagonists (nutlins and related small molecules) is that they operate by occupancy of the p53-binding pocket and, paradoxically, can intensify the intrinsic p53-MDM2 transcriptional feedback loop: p53 activation induces MDM2 expression, increasing the very inhibitor “sink” that must remain saturated to sustain pathway reactivation. Protein degraders reframe this pharmacology by enforcing an acute, knockdown-like removal of MDM2, thereby dampening feedback-driven MDM2 rebound and shifting the system from chronic target coverage to a “hit-and-run” commitment to cell fate (cell-cycle arrest vs apoptosis). In AML-relevant contexts, the VHL-recruiting MDM2 degrader MS3227 exemplifies this principle: it was engineered from an AMG-232–derived MDM2 ligand and optimized through linker structure–activity relationships (alkyl linkers of sufficient length enabled degradation, whereas PEG-type linkers were ineffective), highlighting how ternary-complex geometry and productive ubiquitination—rather than binding affinity alone—dictate degrader performance.

Mechanistically, MS3227 produced rapid, proteasome- and cullin–E3–activity–dependent depletion of MDM2 (blocked by proteasome inhibition or neddylation blockade) and displayed hallmark PROTAC behaviors, including a hook effect at high concentrations consistent with nonproductive binary complex formation [53]. In TP53–wild-type leukemia lines, MS3227 drove stronger p53 pathway output than the matched stoichiometric inhibitor (AMG-232), with more pronounced induction of canonical p53 targets (e.g., p21/CDKN1A, PUMA/BBC3) and greater growth inhibition (reported IC50 ~ 50 nM for MS3227 vs ~250 nM for AMG-232 in MOLM-13), alongside evidence for more sustained pharmacodynamic impact after washout—features consistent with catalytic degradation and altered feedback kinetics in the p53–MDM2 circuit. Importantly for translational positioning, MS3227 activity extended to primary AML specimens in stromal co-culture, where it preferentially reduced leukemic blast populations over more immature CD34⁺ compartments in selected samples and showed limited activity in TP53-deleted disease—reinforcing that, while MDM2 can be degraded biochemically irrespective of TP53 genotype, therapeutic dependency remains strongly enriched in TP53–wild-type settings [53].

The field’s most advanced preclinical dataset to date is arguably provided for KT-253 (Table 1), a highly potent CRBN-recruiting heterobifunctional MDM2 degrader designed explicitly to outperform small-molecule inhibitors by preventing acute MDM2 feedback accumulation and enabling intermittent dosing [54]. In cellular assays, KT-253 achieved subnanomolar MDM2 degradation potency (reported DC50 ~ 0.4 nM in an MDM2-HiBiT system) and translated this into picomolar–subnanomolar functional activity across hematologic models (e.g., RS4;11 growth inhibition IC50 ~ 0.3 nM), while comparator SMIs (including DS-3032/milademetan) induced MDM2 upregulation rather than depletion and were orders of magnitude less potent in matched assays. This potency was coupled to rapid pathway engagement: KT-253 stabilized p53 with very high apparent efficiency (reported EC50 ~ 0.05 nM for p53 stabilization in a mesoscale assay) and induced a broad transcriptional p53 program (including MDM2, GDF15, CDKN1A, GADD45A, TNFRSF10B, FAS, BBC3) at concentrations where DS-3032 produced only modest effects. A key conceptual advance is the demonstration that brief exposure can be sufficient for irreversible apoptotic commitment: short treatment followed by washout still produced caspase activation over 24–48 h, supporting intermittent schedules that may expand the therapeutic window relative to continuous SMI dosing. In vivo, this “pulse pharmacology” translated into striking xenograft efficacy. A single intravenous dose of KT-253 (3 mg/kg) produced complete responses in 5/6 animals in an MV4;11 AML xenograft model, whereas clinically relevant DS-3032 schedules produced no complete responses in that head-to-head setting. Beyond tumor-volume endpoints, the paper reports robust p53 pathway activation and apoptotic execution (cleaved caspase-3) following KT-253 exposure compared with exposure-matched SMI dosing. Clinically relevant combination logic is also emerging: KT-253 synergized with venetoclax in AML models, and in MOLM-13 xenografts a single KT-253 dose combined with venetoclax regimens yielded durable complete responses, whereas either agent alone did not achieve CRs in that resistant model context. Consistent with this axis, the MS3227 study likewise observed venetoclax cooperation associated with MCL-1 downregulation, a mechanistic rationale for overcoming a common venetoclax resistance node in AML [54].

From a development standpoint, these programs also illuminate the core design trade-offs for MDM2 degraders: (i) E3 ligase selection (CRBN vs VHL) to balance tumor expression patterns, depth of degradation, and tolerability liabilities; (ii) linker engineering to optimize productive ternary complex formation (and to avoid hook effects at supraphysiologic exposures); and (iii) selectivity/neosubstrate risk, particularly for IMiD-like CRBN recruiters where off-target neosubstrate degradation is a class concern (albeit KT-253’s deep proteomics profiling in RS4;11 showed high apparent selectivity with p53-pathway upregulation dominating the response signature). Collectively, the preclinical evidence supports the view that next-generation MDM2 degraders may enable more durable pathway reactivation with less continuous exposure, potentially widening the therapeutic index compared with SMIs—especially in TP53–wild-type myeloid malignancies where apoptotic “commitment” after short, high-intensity target depletion appears achievable.

Finally, recent congress reports emphasize the same differentiating themes—feedback-loop suppression, potency versus multiple clinically explored SMIs (e.g., RG7388/idasanutlin, SAR405838, HDM201, AMG-232), and the rationale for intermittent dosing strategies and biomarker-selected indications (including AML).
